# The First United Arab Emirates National Representative Birth Cohort Study: Study Protocol

**DOI:** 10.3389/fped.2022.857034

**Published:** 2022-04-07

**Authors:** Sharon Mutare, Jack Feehan, Leila Cheikh Ismail, Habiba I. Ali, Lily Stojanovska, Abdullah Shehab, Howaida Khair, Raghib Ali, Nahla Hwalla, Samer Kharroubi, Andrew P. Hills, Michelle Fernandes, Ayesha Salem Al Dhaheri

**Affiliations:** ^1^Department of Nutrition and Health, College of Medicine and Health Sciences, United Arab Emirates University, Al Ain, United Arab Emirates; ^2^Institute for Health and Sport, Victoria University, Melbourne, VIC, Australia; ^3^Department of Clinical Nutrition and Dietetics, College of Health Sciences, Research Institute of Medical and Health Sciences, University of Sharjah, Sharjah, United Arab Emirates; ^4^Nuffield Department of Women’s and Reproductive Health, University of Oxford, Oxford, United Kingdom; ^5^Public Health Research Centre, New York University, Abu Dhabi, United Arab Emirates; ^6^Faculty of Agriculture and Food Sciences, American University of Beirut, Beirut, Lebanon; ^7^School of Health Sciences, College of Health and Medicine, University of Tasmania, Launceston, TAS, Australia; ^8^MRC Lifecourse Epidemiology Centre and Human Development and Health Academic Unit, Faculty of Medicine, University of Southampton, Southampton, United Kingdom; ^9^Nuffield Department of Women’s & Reproductive Health, John Radcliffe Hospital, University of Oxford, Oxford, United Kingdom

**Keywords:** nutrition, pregnancy, feeding practices, development, United Arab Emirates

## Abstract

**Background:**

In recent years, the prevalence of non-communicable diseases (NCDs) has escalated. Evidence suggests that there are strong associations between nutrition in early life and the risk of disease in adulthood. This manuscript describes the study protocol of the First United Arab Emirates National Representative Birth Cohort Study (UAE-BCS), with the objective of investigating nutrition and lifestyle factors in the first 1,000 days of life. The main aims of the study are (1) to address critical issues relating to mother and child nutrition and their effect on growth and development, (2) to profile maternal nutrition, child growth, health, and development outcomes in early life, and (3) to study the associations between these factors among the Emirati population in the UAE.

**Methods/Design:**

In this study, a multidisciplinary team of researchers was established including credible researchers from the UAE, Lebanon, Australia, and the United Kingdom to launch the First United Arab Emirates 3-year birth cohort study. We aim to recruit 260 pregnant Emirati women within their first trimester, which is defined by the study as from 8 to 12 weeks pregnant, from obstetrics and gynecology clinics in the UAE. Participants will be recruited via face-to-face interviews and will receive a total of 11 visits with 1 visit in each trimester of pregnancy and 8 visits after delivery. Maternal data collection includes, socio-demographic and lifestyle factors, dietary intake, anthropometric measurements, physical activity, maternal psychological state, and blood samples for biochemical analysis. Post-partum, visits will take place when the child is 0.5, 4, 6, 9, 12, 18, and 24 months old, with data collection including infant anthropometric measurements, young child feeding practices, dietary intake, supplement use and the eating environment at home, as well as all maternal data collection described above, apart from blood samples. Additional data collection for the child includes early child developmental assessments taking place at three timepoints: (1) within 2 weeks of birth, (2) at 10–14 months and (3) at 22–26 months of age. Early child developmental assessments for the infant include vision, hearing, cognition, motor skills, social-emotional reactivity, neurodevelopmental, and sleep assessments.

**Discussion:**

The United Arab Emirates Birth Cohort study protocol provides a standardized model of data collection methods for collaboration among the multisectoral teams within the United Arab Emirates to enrich the quality and research efficiency in early nutrition, thereby enhancing the health of mothers, infants, and children.

## Introduction

Globally the impact of NCDs continues to grow, and they have become the leading cause of mortality, with type T2DM being the fourth most deadly NCD (1, 2). During the past three decades, the global prevalence of T2DM has doubled and now presents a significant public health challenge in almost every country. In 2019, the International Diabetes Federation (IDF) estimated the global prevalence of T2DM to be 9.3%; however, it is thought that around 50.1% of people with diabetes remain undiagnosed. Additionally, the prevalence is estimated rise to 10.2% by 2030 resulting in 115 million new cases between 2019 and 2030. The Middle East and North Africa (MENA) region is ranked the highest region for the prevalence of diabetes accounting for 54.9 million of the global prevalence (3), the United Arab Emirates (UAE) part of the three countries with the highest prevalence of diabetes in the MENA region with 12.3% of the population living with the disease (4, 5). In the UAE, four NCDs are responsible for 65% of all deaths; cardiovascular disease, cancer, diabetes, and chronic respiratory disease (6). A 2010 study in the UAE showed the prevalence of prediabetes to be 31% and undiagnosed diabetes mellitus 14.6%. Another significant health issue is metabolic syndrome (MetS), the concurrent existence of prediabetes, dyslipidemia, elevated blood pressure, and obesity (7).

It is widely acknowledged that most NCDs identified in adults have their origins in early life. The theory of the developmental origins of adult diseases, commonly known as the Barker hypothesis, associates nutrition during pregnancy, and risk of chronic disease in later life (8). Accumulating evidence shows that a balanced and varied diet plus a healthy lifestyle in the months leading up to conception, throughout pregnancy, during infancy, and to the second birthday, collectively known as the first 1,000 days of life, are influential to health and risk of disease later in life. For instance, full-term infants who are small-for-gestational age have an increased risk of cardiovascular disease (CVD) and T2DM in adulthood, and this is a known consequence of fetal undernutrition (9). Studies on the relationships of nutritional imbalances in early life with birth outcomes, growth patterns, and early determinants of NCDs, have revealed direct associations between limited maternal weight gain during pregnancy and both impaired fetal growth, and low birth weight (10). Also, excess maternal weight gain correlates with high birth weight and fetal growth (large-for-gestational age) (11, 12), as well as adverse cardiometabolic profiles in the offspring (13). In addition to maternal weight gain, maternal diet composition and micronutrient status during pregnancy seem to be associated with birth outcomes and child health status.

The first 1,000 days of life are a vital period that play a crucial role in the development of the immune, endocrine, metabolic, motor, and other developmental pathways. Maternal nutrition and lifestyle choices in preconception and during pregnancy have significant influences on the health, growth, and development of the fetus. Research shows that a maternal diet low in protein is connected to changes in the structure of the liver of the fetus and low birth weight (14), while a diet rich in fat effects adipocyte metabolism, fetal growth, and fat mass in the offspring (15, 16). Maternal anemia and iron, folate, or vitamin B12 deficiency during pregnancy is associated with an increased risk of fetal growth restriction, premature birth, and low birth weight (17–19). The effect of maternal nutrition on the cognitive capability and neurodevelopment of offspring is well established, particularly in the context of micronutrient deficiencies. Vitamin B12 deficiency is associated with spina bifida and infant insulin resistance (20), and maternal iodine deficiency with hypothyroidism. Prenatal maternal iron deficiency is associated with decreased myelination, disturbance in monoamine neurotransmitter synthesis and hippocampal energy metabolism in offspring (21), and there is evidence of an association between increased consumption of essential long-chain omega -3 fatty acids during pregnancy and better infant performance in development assessments, verifying the link between prenatal and postnatal diet to specific aspects of the brain development (22). The effect of prenatal maternal nutrition on neurodevelopment outcomes in children is not exerted solely through the effect of maternal nutrition on fetal growth and birth outcomes. It persists into the postnatal period, by impacting the quality and quantity of breast milk, maternal mood, and infant feeding practices.

Maternal and infant nutrition may influence the risk of later disease due to its critical associations with immunological development of the fetus and young infant (23). Adequate nutrition is necessary both for establishment of the immune system, normal organogenesis and development, and development of an adequate immune response. Undernutrition during early childhood exerts a concomitant effect on cognitive and developmental milestones through mid- and late- childhood and into adolescence. Studies consistently identify associations between stunting at ages 2 and 3 years with later cognitive deficit, as well as school achievement, even after controlling for socio-demographic confounders (24).

Global estimates of stunting and wasting in children under the age of five for 2018 were reported to be 21.9% (149 million) and 7.3% (49 million), respectively, and obesity in children of the same age group was reported to be 5.9% (40 million). The MENA region has the second highest rate of under 5 years overweight children, with an increase from 8.9% in 2,000 to 11.2% in 2018 (25). According to the UNICEF, in 2020 the UAE mortality rate for children under five was 6.6 per 1,000 live births, for infants was 5.6 per 1,000 live births, and neonatal mortality rate was 3.6 per 1,000 births (26). The percentage of overweight school and adolescent children (5–9 years) was estimated to be 37% in 2016 (27). However, there are no records for malnutrition, stunting and wasting of children in the country.

In some research from the UAE, the overall prevalence of global development delay and pervasive development disorder among 3-year old children was reported to be 2.44% and 29 in 10,000 for severe development delay/disorder with a prevalence of 8.4% and 58 in 10,000 for mild to moderate delay/disorder (28). Currently, there are no reports of the neurodevelopmental abilities of young children during the first 1,000 days of life from the UAE. These indicators are considered key public health factors, which contribute independently to mortality and burden of disease.

### Objectives

The objective of the first UAE -BCS is to recruit 260 pregnant Emirati women and to profile the maternal nutrition, child growth and developmental outcomes during the first 1,000 days of life in the UAE. This 3-year, prospective observational study seeks to address critical issues relating to mother and child nutrition and its effect on growth and development. Specifically, given that maternal and paternal health has a transgenerational impact on the development and health of offspring, this cohort study aims to profile maternal nutrition, child growth and development outcomes, and to study the association between these in the UAE. Accordingly, the establishment of a body of evidence through this cohort study is of paramount importance to inform future strategies regarding optimal dietary intake and consequent nutrition-related health of women in childbearing age and their offspring. The objectives of this cohort study involve:

- Characterization of the *in utero* environment through assessment of antenatal maternal and paternal factors such as, weight gain, abnormal gestational glucose tolerance, pregnancy-induced hypertension, preterm births, and new-born small- and large—for gestational age birth weight on new-born body composition, birth outcomes, and growth patterns.
- Studying the association between early feeding practices including breastfeeding, weaning, and complementary feeding patterns, as well as postnatal growth and adiposity for the first 1,000 days of life.
- Studying the association between specific maternal micronutrient status (vitamin B12, vitamin A, vitamin D, hemoglobin, ferritin, folate, lead, and zinc) and gestational weight gain with offspring body composition, birth outcomes and growth patterns.
- Determining the impact social and environmental conditions, including family structure, socioeconomic status, health behaviors (i.e., dietary intake and physical activity), and maternal psychological stress factors on the development of adiposity of the growing offspring in the first 1,000 days of life.
- Developing and validating the Neonatal Neurodevelopment Assessment (Neo-NDA) for the measurement of neurological status and maturity in new-borns, which will ultimately serve clinical and academic research purposes.

## Methods and Analysis

### Ethical Approval

The UAE-BCS protocol was approved by the United Arab Emirates University Human Research Ethics Committee, (ref. ERH-2020-61442020-12). Additional approval was granted from the Ministry of Health and Prevention (Approval Reference No: MOHAP/DXB-REC/NDD/No.162/2020.) and the Department of Health Abu Dhabi (ref: DOH/CVDC/2021/510). This study is registered at clinicaltrials.gov (NCT04928898).

### Study Design

The UAE-BCS is a prospective, observational, population-based 3-year cohort study of expecting Emirati females and their children living and attending obstetrics and gynecology (OB-GYN) clinics in the UAE. In 2021, the UAE population was reported to be 10 million, however, the expatriates constitute the majority of the population, and the Emirati population was estimated to account for almost 1 million of the entire population in 2010 (29, 30). The UAE has seven Emirates in which the Emirati population is reported to be a s follows; Emirate of Abu Dhabi was estimated to have 551,353 Emiratis in 2016; the Emirates of Dubai was estimated to have 233,430 in the same year, and most recently 271,050 in 2020; Ras Al Khaimah had 127, 000 Emiratis in 2015. However, data for Ajman, Umm Al Quwain and Fujairah estimated 42, 186, 17,482 and 64,860 Emiratis in 2010, respectively. Additionally, in all Emirates males account for most of the Emirati population in all Emirates with the exception of Umm Al Quwain in which 8, 811 of the 17, 482 were female in 2010 (31–34).

For this study, the UAE will be stratified into three geographic areas based on population density and socioeconomic factors: Abu Dhabi, Dubai, and the Northern Emirates. Approximately 40% of participants will be recruited from Kanad Hospital, Al Ain, Abu Dhabi; 30% of participants from facilities in Dubai, and 30% from clinics across the Northern Emirates.

### Study Participants

The study aims to recruit a total of 260 pregnant Emirati females during their first trimester (8–12 weeks of gestation) through face-to-face recruitment at OB-GYN clinics in selected health care facilities across the Emirates of the UAE. The participants will be provided with both verbal and written information (Participant Information sheet) about the study aims, data collection activities, and duration of the study. Mothers will consent to the study on behalf of their children to be included in the study, by signing a form, after meeting the following inclusion and exclusion criteria.

Inclusion Criteria:

• Emirati Nationality
• Within first trimester of pregnancy (between 8–12 weeks of gestation)
• Singleton pregnancy
• Absence of major illness preconception (Diabetes mellitus, hypertension, kidney disease, cancer, epilepsy, severe psychiatric illness, and other chronic diseases of infections such as autoimmune disorders, human immunodeficiency virus, and hepatitis).

Exclusion Criteria:

• Non-Emirati Nationality
• Unconfirmed viable, intrauterine pregnancy at first obstetric ultrasound during first trimester
• Twin or multiple gestation pregnancy and a history of multiple gestations
• History of chronic illness (i.e., T2DM, hypertension, etc.)
• Previously given birth to babies with malformation, intellectual disability, or inborn errors of metabolism.
• Experience of a miscarriage before 28 weeks of gestation
• Use of significant regular medications, including insulin, anti-hypertensive agents, psychotropic medications, anti-epileptic drugs, steroids, immune-suppressive agents, and chemotherapeutic agents.

### Study Protocol

**FIGURE 1:**
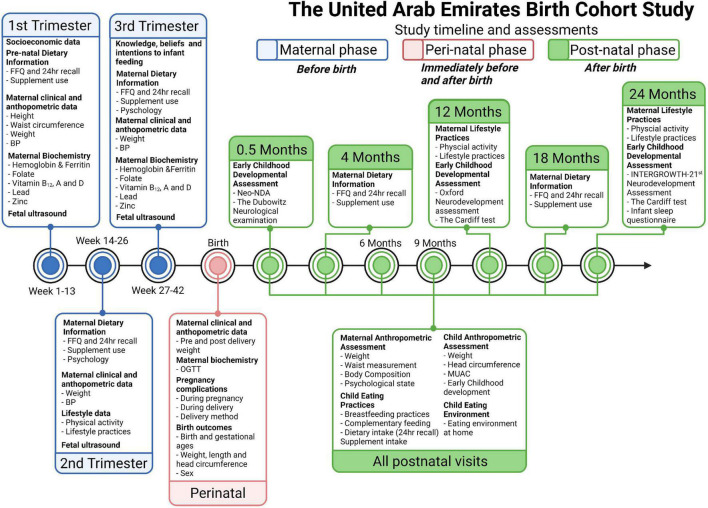
Assessment timeline for the UAE Birth Cohort Study (UAE-BCS).

The study protocol is summarized in Figure 1. Each mother/child will be assessed over 3 years, at 11 separate study visits.

#### During Pregnancy

Pregnant females will be recruited during their first trimester (8–12 weeks of gestation) in OB-GYN clinics and perinatal health facilities in the UAE. There will be one visit during each trimester of pregnancy, and an additional eight visits postpartum. The initial prenatal assessment screening will aim to measure key factors such as diet and supplement use, physical activity and lifestyle practices, maternal psychological, sociodemographic, and socioeconomic characteristics, through anthropometric measurements and validated questionnaires. Blood samples will be collected from participants during the first and third trimester for biochemical analysis of several biomarkers. Maternal exposure, knowledge, attitudes, and intention toward infant feeding practices will be assessed during the third trimester through questionnaires. Information on fetal growth from ultrasound will be collected during the second and third trimester.

#### Post-partum

The participants’ hospital records will be used to obtain information about the delivery, as well as birth outcomes data. Postnatal visits will take place when the child is 0.5, 4, 6, 9, 12, 18, and 24 months old. These visits will consist of anthropometric measurements and assessment of child feeding practices (breastfeeding and complementary feeding practices, dietary intake, supplement use, and the eating environment at home). Developmental assessments will take place at three points: (a) within 2 weeks of birth, (b) at 10–14 months, and (c) at 22–26 months of age. In addition, the mothers will undergo postpartum assessment at each postnatal visit. This will consist of anthropometric measurements, collection of dietary intake data, supplement use, lifestyle habits, physical activity, and psychological state.

#### Data Collection Instruments and Methods

Culture specific and English and Arabic questionnaires were developed for the purpose of data collection for this study. In addition, questionnaires for infant neurological development and neonatal forms were provided by an expert in the field and translated into Arabic. The content and validity of all questionnaires was confirmed by experts in each respective field. A pilot study was conducted to test the wording, cultural appropriateness of the questionnaires, and to ensure they would yield the required data. The questionnaires will be administered by trained research nutritionists through face-to-face interviews.

### Sociodemographic and Lifestyle Questionnaires

This questionnaire will be used to collect sociodemographic information about the parents, including age, education, employment status, income, dietary, and lifestyle influences and choices, and household details including information about living arrangements, such as type of accommodation. Additional paternal information collected in this questionnaire include brief questions about general health.

#### Maternal Dietary Intake Assessment Questionnaire

Maternal dietary intake information will be collected through Food Frequency Questionnaires (FFQ) and multiple pass 24 h food recalls and will be used to assess energy, nutrient intake, and dietary patterns. The FFQs contain 130 culture-specific food items, and supplement use will also be collected during the 24-h recall. The FFQs were specifically created for population studies in the UAE and will include foods traditionally consumed during pregnancy. Additionally, the FFQs will contain questions about the weekly consumption frequency of different foods and food groups. The United States Department of Agriculture Multiple Pass Food Recall (MPFR), which is a 24-h recall method used to collect detailed dietary intakes and supplement use of participants to allow capture of all food and beverage consumption during the 24 h preceding the interview, and is validated for accurate measurement of food intake (35). The 24-h food recalls will be analyzed with the ESHA Food Processor, SQL (ESHA Research, V. 11. 0) to calculate energy and nutrient intake in the participants (36). During the study, both the FFQs and the 24-h recalls will be conducted during the first, second and third trimester, and 4 months post-partum.

#### Physical Activity Questionnaire

A pregnancy-specific physical activity questionnaire used in a similar study in Qatar and Lebanon will be used to assess the physical activity levels of the participants during their second trimester (37). The International Physical Activity Questionnaire (IPAQ) will be used to collect physical activity levels of the mother at each child follow up visit (38).

#### Infant and Young Child Feeding Practices and Dietary Intake

The WHO indicators will be used as benchmarks to assess child breastfeeding and complementary feeding practices and will include questions related to breastfeeding and complementary feeding (39). A 24-h recall using the mother as a proxy for the child’s dietary intake and supplement use will be used for evaluation at each of the visits post-partum.

### Anthropometric Assessments

Anthropometric measurements of the mothers, infants and young children will be obtained at different stages throughout the study.

#### Anthropometric Assessment of Mothers

Maternal anthropometric measurements will be taken during pregnancy and will follow standard techniques for obtaining height in centimeters (cm) using a stadiometer during the first trimester, weight in kilograms (kg) using Tanita scale (Tanita BC-418, Tanita Corp., Tokyo, Japan) at all visits, waist circumference in centimeters using a non-stretchable measuring tape during the first trimester and percentage body fat which will be assessed by bioelectrical impedance analyzer (Tanita BC-418, Tanita Corp., Tokyo, Japan) during the first trimester and during all postnatal visits.

#### Anthropometric Assessment of Infants and Young Children

Anthropometric assessment of infants and young children will be taken at each of the six visits after birth and will include measurement of weight, length, head circumference and mid-upper arm circumference (MUAC). Anthropometric measurement protocols and quality control procedures will follow the WHO MGRS INTERGROWTH-21st Project protocol (40–42). Study equipment will include portable electronic weighing scales (Seca model 376; Seca, Hangzhou, China); recumbent length will be measured using a Harpenden Infantometer (range 300–1,100 mm; Chasmors Ltd., London, United Kingdom), flat metal tape measure will be used for head circumference measurement (CMS ref.3105; Chasmors Ltd., London, United Kingdom). MUAC will be measured using a tape around the arm at the level of the midpoint (42).

#### Early Child Development Assessments

For this study, a set of formal validated questionnaires and developmental assessment tools will be used to assess the child’s growth milestones and development, such as vision, hearing, attainment of certain reflexes and motors kills, social-emotional behaviors, and language.

Assessment of early child development (ECD) will occur at three timepoints: (i) within 2 weeks of birth; (ii) at 10–14 months of age; and at 22–26 months.

The first developmental assessment will take place within 2 weeks and consist of a brief examination of the newborn for color, posture, tone, reflexes, suck, behavior and dysmorphic features using a novel tool, the Neonatal Neurodevelopment Assessment (Neo-NDA). In a sub-sample of 30 participants, the Dubowitz’s neurological examination (43), a clinical examination of neonatal tone, reflexes and behavior will be administered together with the Neo-NDA.

The second developmental assessment will occur at 10–14 months of age, using the Oxford Neurodevelopment Assessment (OX-NDA). The OX-NDA is a 57-item measure and assesses nine domains: cognition (15 items); fine and gross motor (8 items); language (8 items); behavior (7 items); executive function (3 items); attention (5 items); social-emotion reactivity (9 items); and positive affect (2 items). The instrument has a combination of directly administered, concomitantly observed, and maternally reported items. The child’s performance on each item is reported on a five-point scale. The OX-NDA was designed to be an international, population-based screening measure for assessing early child neurodevelopment without the need for specialist professionals or infrastructure and has been validated against the Bayley Scales of Infant Development in a sample of 522 from Pelotas, Brazil (44). Hearing will be assessed by mapping 11 items on the OX-NDA onto 4 auditory milestones for the 10–14 months age group (45). Vision outcomes in the infants will be evaluated using the Cardiff Tests, which measure visual acuity and contrast sensitivity (46, 47). Mothers will report on the sleep of their infants using a modification of the brief infant sleep questionnaire (48).

The final developmental assessment will occur at 22–26 months of age, using the INTERGROWTH-21st Neurodevelopment Assessment (INTER-NDA) (49, 50). The INTER-NDA is a 53-item assessment of cognition, expressive and receptive language, gross and fine motor skills, behavior, attention, and social-emotional reactivity in children 2 years old. Outcomes are reported on a 5-point scale characterizing the child’s performance in each domain across a spectrum. Like the OX-NDA, it designed to be free from cultural biases and is based upon objective reporting (rather than subjective judgment) of the child’s performance. It consists of directly administered, concurrently observed, and caregiver reported items. It’s inter-rater and test-retest reliability are *k* = 0.70, 95% CI: 0.47–0.88 and *k* = 0.79, 95% CI: 0.48–0.96, respectively (49), and the INTER-NDA shows good agreement with the Bayley Scales of Infant Development III edition (intraclass correlations for domains ranging between 0.745 and 0.883; *p* < 0.001). The INTER-NDA has been used to assess ECD outcomes in over 5,000 children from 12 countries.^1^ Hearing will be assessed by mapping 6 items on the INTER-NDA onto 3 auditory milestones for the 22–26 months age group (45). Vision outcomes in the infants will be assessed using the Cardiff Tests, and mothers will report on infant sleep outcomes on a modification of the brief infant sleep questionnaire.

### Biochemical and Blood Pressure Assessments

During pregnancy, fasting blood samples will be obtained from the mother twice, in the first and third trimester to assess maternal micronutrient status. The blood samples will be collected by a certified phlebotomist into appropriate test tubes (with or without ethylenediaminetetraacetic acid (EDTA) appropriate for the biomarker to be analyzed. Iceboxes will be used to store the test tubes temporarily until ultimate centrifugation and analysis. The following are the biomarkers that will be examined and with corresponding analytical method:

- Hemoglobin (HemoCue^®^ Glucose 201 + System)
- Ferritin (Electrochemiluminescent assay, RocheCobas E 411; Roche Diagnostics, Indianapolis, IN, United States)
- Folate (Electrochemiluminescent assay, Roche Cobas E 411)
- Vitamin B12 (Electrochemiluminescent assay, Roche Cobas E 411)
- Vitamin A (High-Performance Liquid Chromatography)
- Vitamin D (Electrochemiluminescent assay, Roche Cobas E 411)
- Lead [Inductive Captured Plasma (ICP)]
- Zinc [Inductive Captured Plasma (ICP)]

Blood pressure will be measured with a validated and calibrated digital automated sphygmomanometer (Omor Hem-907, Omron Healthcare, Kyoto, Japan) with participants seated after a 5-min rest. These measurements will be obtained at the three visits during pregnancy.

#### Delivery and Birth Outcome Data

Delivery and birth outcome data will be obtained from the records of the hospital at which the birth takes place with the participant’s consent. The data includes occurrence of complications during pregnancy, date and method of delivery, gestational age, sex of newborn, birthweight, length, and head circumference all of which will be measured by a trained researcher or clinician.

### Reliability of Data Collection

The questionnaires to be used in the study were reviewed by the experts in the field. Additionally, the validity of questionnaires will be tested using a pilot group of the study population, however, the FFQ and IPAQ have been validated and used in the UAE population in recent studies (51, 52). Considering that the data will be collected by trained research assistants across the different states of the UAE, several quality assurance activities will be implemented to ensure consistency and reliability standards of the collected information. The principal investigators have compiled a data collection training program with a certification process and detailed operations manual describing the procedures before, during and after data collection to be given to each research assistant. The manual will contain checklists to be followed before and after each visit, including a section on Frequently Asked Questions (FAQs). The instruments used in data collection were carefully chosen and will be identical in each of the facilities where data will be collected, such as measuring tapes, stadiometers, scales, etc. Additionally, there will be regular meetings between the principal investigators and personnel involved in data collection in which probing and interviewing techniques will be standardized to minimize interviewer bias. The principal investigators will regularly visit participating healthcare centers to observe data collection activities, discuss emerging situations and ensure that all procedures are standardized.

### Statistical Analysis and Sample Size Calculation

The data collected will be checked for accuracy and completeness and will be entered into the Statistical Package for Social Science (SPSS) software, using the most current version. The primary aim of the study is to examine the associations between maternal nutrition (e.g., weight gain, micronutrient status, etc.) and infant health outcomes (e.g., growth rate, dietary diversity score, feeding practice score, etc.). Given the somewhat limited data on the above-mentioned relationships, for power calculation an *r* = | 0.2| was chosen, as it yields the largest sample size. Using the UCSF sample size calculator, the sample size needed to detect a correlation as small as | 0.2|, at 80% power and 5% type I error, is determined at 194. For this study, correlations less than 0.20 would be too small to be considered clinically significant (37). Given the high attrition rate seen in previous birth cohorts (37), we estimate that roughly 15% of the participants will drop out after the first visit. An additional 10% was factored into for losses due to preterm delivery or still birth. More participants (∼10% of sample) are also required to test for simple interactions in our study (e.g., Emirati, Sex, and Timing). Thus, the original sample size was increased by 35% and the intended sample size was determined at 260 pregnant women.

Frequencies and percentages will be used to describe categorical variables and mean, and standard deviations will be used to describe continuous variables. The chi-square test (χ2) or Fisher’ s exact test will be used to calculate the association between categorical variables. Independent *t*-tests and Mann-Whitney tests will be used to chart comparisons for normal and non-normal continuous variables, with normality of variables evaluated using the Kolmogorov-Smirnov and Shapiro-Wilk tests. Associations among the variables of the cohort study (i.e., maternal factors, birth outcomes, breastfeeding, complementary feeding, growth patterns, etc.) will be examined using simple linear and logistic regressions. The two main dependent variables in this study are related to birth outcomes and growth patterns of the child. Variables showing statistical significance in the univariate analysis will be included in the multivariate models as covariate factors in order to control for potentially confounding variables that have been used in the model. Parameter estimates and their respective 95% confidence intervals will be calculated for linear regression analyses, whilst for logistic regression analyses odds ratios and their respective 95% confidence intervals will be calculated. Tests for linearity (tolerance > 0.4) of the covariates included in the regression models will be performed. Normality of the residuals will be assessed using the histogram of standardized residuals and normal probability plot in all linear regression models. *P*-values less than 0.05 will be considered to indicate statistical significance.

## Discussion

The design for the 3-year birth cohort study of pregnant Emirati women and their offspring has been thoroughly described in this study protocol. The study investigates relationships between nutrition and lifestyle factors with birth and growth outcomes in the UAE and explores the early determinants of NCDs. Existing studies of the UAE population has focused mainly on the determinants of obesity and NCDs in adults, with some studies focusing on investigating the prevalence of obesity and its determinants in adolescent children. There is a scarcity of detailed research on the dietary practices during pregnancy or infant and young child feeding practices in the UAE with previous studies mainly cross sectional in design, with small sample sizes which are not nationally representative (53–56).

Research has established that growth and development in early life is crucial to longer-term health status, wellbeing, and behavior across the lifespan (57). Nutrition and lifestyle behaviors are similarly critical for maternal and offspring health from preconception, through pregnancy and into early postnatal life (58). Given the rapidity of the development of the obesity epidemic and increased prevalence of related NCDs, particularly in the UAE, the changes cannot be explained by genetics alone but rather in key environmental factors (59, 60). Accordingly, optimal nutrition during pregnancy has important implications for fetal development and growth and may be protective for the long-term health outcomes of both the mother and child. Prenatal genetic and environmental factors that can affect the first 1,000 days of life have recently been shown to play a pivotal role in early childhood development including cognitive, social-emotional skills and motor development. Moreover, the global double burden of malnutrition is increasing, and the UAE lacks data for malnutrition, stunting and wasting of children. Undernutrition during early childhood exerts a concomitant independent effect on the cognitive and developmental abilities developing through mid- and late-childhood and into adolescence (61). Prospective cohort studies consistently show significant associations between stunting at age 2 or 3 years and later cognitive deficits, school achievement, and dropout, after controlling for socio-demographic confounders (24).

The overall prevalence of global developmental delay and pervasive developmental disorder among 3-year-old children in the UAE in 2016 was reported to be 2.44% and 29/10,000 for severe delay/disorder with a prevalence of 8.4% and 58/10,000 for mild to moderate delay/disorder, respectively, with no reports profiling the neurodevelopment abilities of young children during the first 1,000 days of life from UAE (28). Therefore, the importance of the first few years of a child’s life to decreasing the risk of disease morbidity, mortality, and later risk of chronic disease, and to promoting healthy growth and development, is important. Moreover, nutrition during the first 1,000 days of a child’s life has been identified as one of the significant modifiable factors, during this critical window of opportunity, to influence optimal growth, promote optimal neurodevelopment and reduce disease vulnerability later in life (61). In addition, the data obtained in this study will support the development and validation of a new tool, the Neo-NDA, to be used for the measurement of neurological status and maturity of new-borns in the UAE; explore the association between early childhood development (ECD) outcomes using the three inter-related tools: Neo-NDA, the OX-NDA and the INTER-NDA at < 5, 12, and 24 months of life; and use holistic and standardized measures of ECD, importantly including measure of vision, hearing and sleep at all developmental assessments. Prenatal maternal nutrition is an important predictor of fetal and child brain development, exerting its effect in both intrauterine (fetal growth and birth outcomes) and extrauterine (parenting, child feeding practices, maternal mood, quality, and quantity of breast milk) environments. There is limited understanding on what constitutes optimal ECD, with most studies employing heterogeneous methodologies for ECD assessment and being carried out in high-income western settings. This has limited both the comparability and generalizability of neurodevelopmental evaluations, between studies, and to other populations. It also restricts their scalability for measuring and monitoring ECD at a population level with routine ECD screening programs lacking in many parts of the world. To our knowledge, there are no studies profiling neurodevelopmental outcomes during the first 1,000 days of life in the UAE, and therefore our findings will fill this gap. There is a need for high quality data to be collected in country/geography-specific large birth cohorts, using standardized methodologies, to guide understanding of what constitutes optimal and sub-optimal child development in these contexts, to permit comparability between populations, representing a scalable solution to the application of ECD surveillance in these settings. Birth cohort studies that commence either during pregnancy or preconceptionally and then follow the offspring and the wider family into later life, can make a significant contribution to the development of dietary recommendations to expectant mothers during pregnancy to maximize the health and development of the offspring while simultaneously contributing to the understanding of optimal and sub-optimal ECD outcomes in the context of maternal nutrition. Maternal and infant nutrition, as well as early child growth, health, and development, should be a priority for health policy implementation, health promotion activities, and interventions.

The findings of this study will not only provide evidence and data for the UAE population that has never been collated before, but it will also survey infant and maternal body composition and contribute to reproductive health policy. In addition to laying the foundations for culture specific health policies and interventions for pregnant women, infants and young children, this study will provide the first database for the UAE and highlight gaps in the current evidence while identifying avenues for further research. The hallmark of the UAE birth cohort study protocol includes intersectoral collaborations and a versatile research team, and the comprehensive assessment of maternal and infant nutrition and lifestyle exposures. An important aspect of the study is the collaboration between OBYGYN clinics and selected health care facilities across the UAE with a panel of experts and research personnel in this study, particularly in recruitment, data collection and follow up. Importantly, the team of researchers involved in this study are experts in obstetrics, neonatal development, and nutrition epidemiology. Another key aspect of the study is the methodical observation, monitoring and examination of dietary intake lifestyle practices, body composition, and growth of mothers and their off springs from conception to 2 years of after birth.

## Conclusion

The UAE-BCS will provide a key platform of evidence and skill to inform future strategies to optimize dietary and consequent nutrition-related health of women in child-bearing age and their children, and thereby, the health of the nation. It will also provide critical data to inform policy decisions, and public health interventions in the region, providing a path to reduce the growing impact of obesity and NCDs in the UAE and MENA region more broadly.

## Author Contributions

LS, AA, LC, AH, HA, MF, NH, RA, AS, and HK: conceptualization. SM and JF: writing—original draft preparation. LS, AA, LC, AH, HA, SK, MF, NH, RA, and AS: writing—review and editing. JF: visualization. LS and AA: supervision. All authors contributed to the article and approved the submitted version.

## Conflict of Interest

The authors declare that the research was conducted in the absence of any commercial or financial relationships that could be construed as a potential conflict of interest.

## Publisher’s Note

All claims expressed in this article are solely those of the authors and do not necessarily represent those of their affiliated organizations, or those of the publisher, the editors and the reviewers. Any product that may be evaluated in this article, or claim that may be made by its manufacturer, is not guaranteed or endorsed by the publisher.

## Footnotes

1
www.inter-nda.com
